# Handedness in schizophrenia and affective disorders: a large-scale cross-disorder study

**DOI:** 10.1007/s00406-024-01833-9

**Published:** 2024-06-25

**Authors:** Annakarina Mundorf, Alexander Lischke, Jutta Peterburs, Nina Alexander, Linda M. Bonnekoh, Katharina Brosch, Kira Flinkenflügel, Janik Goltermann, Tim Hahn, Andreas Jansen, Susanne Meinert, Igor Nenadić, Navid Nico Schürmeyer, Frederike Stein, Benjamin Straube, Katharina Thiel, Lea Teutenberg, Florian Thomas-Odenthal, Paula Usemann, Alexandra Winter, Udo Dannlowski, Tilo Kircher, Sebastian Ocklenburg

**Affiliations:** 1 ISM Institute of Systems Medicine & Department of Human Medicine, MSH Medical School Hamburg, Am Kaiserkai 1, 20457 Hamburg, Germany; 2 Department of Neurology, Division of Cognitive Neuroscience, Johns Hopkins University School of Medicine, Baltimore, MD USA; 3 Department of Psychology, MSH Medical School Hamburg, Hamburg, Germany; 4 ICPP Institute for Clinical Psychology and Psychotherapy, MSH Medical School Hamburg, Hamburg, Germany; 5 Department of Psychiatry and Psychotherapy, University of Marburg, Marburg, Germany; 6 Center for Mind, Brain and Behavior (CMBB), University of Marburg and Justus Liebig University, Giessen, Germany; 7 Institute for Translational Psychiatry, University of Münster, Münster, Germany; 8 Department of Child and Adolescent Psychiatry, Psychosomatics and Psychotherapy, University of Münster, Münster, Germany; 9 Institute of Behavioral Science, Feinstein Institutes for Medical Research, Glen Oaks, USA; 10 Core-Facility Brainimaging, Faculty of Medicine, University of Marburg, Marburg, Germany; 11 Institute for Translational Neuroscience, University of Münster, Münster, Germany; 12 ICAN Institute for Cognitive and Affective Neuroscience, MSH Medical School Hamburg, Hamburg, Germany; 13 Institute of Cognitive Neuroscience, Biopsychology, Faculty of Psychology, Ruhr University Bochum, Bochum, Germany

**Keywords:** Laterality, Hemispheric asymmetries, Depression, Bipolar, Schizoaffective, FOR2107

## Abstract

While most people are right-handed, a minority are left-handed or mixed-handed. It has been suggested that mental and developmental disorders are associated with increased prevalence of left-handedness and mixed-handedness. However, substantial heterogeneity exists across disorders, indicating that not all disorders are associated with a considerable shift away from right-handedness. Increased frequencies in left- and mixed-handedness have also been associated with more severe clinical symptoms, indicating that symptom severity rather than diagnosis explains the high prevalence of non-right-handedness in mental disorders. To address this issue, the present study investigated the association between handedness and measures of stress reactivity, depression, mania, anxiety, and positive and negative symptoms in a large sample of 994 healthy controls and 1213 patients with DSM IV affective disorders, schizoaffective disorders, or schizophrenia. A series of complementary analyses revealed lower lateralization and a higher percentage of mixed-handedness in patients with major depression (14.9%) and schizophrenia (24.0%) compared to healthy controls (12%). For patients with schizophrenia, higher symptom severity was associated with an increasing tendency towards left-handedness. No associations were found for patients diagnosed with major depression, bipolar disorder, or schizoaffective disorder. In healthy controls, no association between hand preference and symptoms was evident. Taken together, these findings suggest that both diagnosis and symptom severity are relevant for the shift away from right-handedness in mental disorders like schizophrenia and major depression.

## Introduction

Handedness is the most widely investigated form of behavioral lateralization and can be differentiated into hand preference and hand skill [1]. Interestingly, studies systematically investigating associations between different lateralization measures and data on mental well-being, stress reactivity, and sub-clinical symptoms highlight that especially hand preference shows high correlations with several measures of mood and stress reactivity in the general population [2, 3]. Notably, hand preference refers to the preferential use of one hand for specific tasks, such as writing or throwing a ball. The preferred or dominant hand is faster and more accurate in performing these tasks than the nondominant hand [4]. Generally, there are four different forms of handedness: right-, left-, mixed-handedness, and ambidexterity [4]. Right- and left-handed individuals clearly prefer the right or left hand and thus perform most tasks with their dominant hand. Mixed-handed individuals perform some tasks better with the left hand and some with the right hand, whereas ambidextrous individuals perform a tasks such as writing equally well with both hands, which is rare (3.3%) [5]. The prevalence of non-right-handedness is almost double in twins, with 11.11–16.19% [6, 7] compared to 7.23% in singletons [7]. Moreover, an increase in neurodevelopmental delays and higher externalizing problems were reported in mixed-handed twins in a study with over 35.000 Dutch twins [6]. In clinical samples, meta-analyses revealed significantly increased rates of non-right-handedness for attention deficit hyperactivity disorder [8], autism spectrum disorders [9], posttraumatic stress disorder [10], and schizophrenia [11].

The preference of one hand over the other is a behavioral result of structural and functional left-right differences in the brain: so-called hemispheric asymmetries [1]. These hemispheric differences are inherent, found across species, and may increase brain efficiency [12–14]. In humans, over 90% of cortical and subcortical regions demonstrate structural asymmetry [15, 16]. Given that structural and functional asymmetries are present in core structures involved in emotion processing, such as the amygdala [17, 18], it is hypothesized that altered asymmetries in such regions may result in altered emotion processing as seen in mental disorders. In line with this, changes in amygdala volume or shape have been reported in subjects with depression [19, 20] and schizophrenia [21]. So far, many neurodevelopmental and mental disorders have been associated with altered functional hemispheric asymmetries in regions related to cognitive functions that are associated with the respective psychopathology [22].

Generally, hand preference is determined before birth [23, 24] whereas the strength of lateralization, that is, how strongly one prefers the right or left hand, may vary throughout life [25]. Thus, it is hypothesized that somehow non-specific or specific factors influence atypical lateralization (e.g., non-right-handedness) and psychopathological symptoms [22] but that, e.g., non-right-handedness is present first, whereas psychopathological symptoms slowly develop over time. In line with that, a study including healthy children found higher mental health problems in left-handed compared to right-handed children [26].

Admittedly, to date, it is not known in what way increased rates of non-right-handedness and mental disorders are linked to one another. On a theoretical level, three different types of associations can be conceived to explain the found association [22]:

- **Non-specific association**: Non-specific factors influence non-right-handedness and the general risk of developing any type of mental disorder. Therefore, different disorders should exhibit similar rates of non-right-handedness.
- **Diagnosis-specific association**: Specific factors affect both non-right-handedness and the risk for a specific disorder. These factors would also be functionally specific, i.e., related to neural networks linked to a particular disorder with little overlap between disorders.
- **Symptom-specific association**: Factors related to a symptom group, such as cognitive or affective problems, influence both non-right-handedness and psychopathology. This symptom group is independent of a specific diagnosis. Consequently, non-right-handedness would be linked to specific symptoms with increased severity but independent of a diagnosis.

Results from genetic studies support the non-specific association. For example, a genome-wide association study on handedness found positive correlations between genetic variants associated with left-handedness and neuropsychiatric traits, such as schizophrenia and bipolar disorder [27]. The study thus supports a genetic predisposition for both, left-handedness and some psychiatric disorders [27]. Cross-disorder findings further support this. For example, a large-scale neurogenetic study indicated that genetic variants associated with gray matter brain asymmetry overlapped with genetic variants associated with autism spectrum disorder and schizophrenia [28]. Given the reported genetic overlap between autism spectrum disorder and schizophrenia [29], these results appear to suggest that factors not specific to either disorder may be the driving forces.

Support for the diagnosis-specific association especially comes from studies on language-related disorders. Here, genetic variants of genes related to language networks were linked to altered functional brain asymmetry [30]. In line with that, higher rates of non-right-handedness have been confirmed in children with language and/or reading impairments by meta-analysis [31]. However, altered asymmetries in brain regions relevant to language processing have also been found in patients with schizophrenia [32], instead supporting that atypical asymmetries may be linked to symptoms rather than to a specific diagnosis.

The symptom-specific approach is, for example, supported by studies in patients diagnosed with depression or schizophrenia. Here it seems like some symptoms are associated with altered lateralization, whereas other symptoms are not. Research on the association between depression and non-right-handedness suggests that similar to results found in schizophrenia, it may be possible that not all but only some symptoms are associated with non-right-handedness in depression. For example, a large population-based sample of individuals aged 50 years and older from 12 European countries assessed depressive symptoms using three different inventories. Overall, they found increased depressive symptoms in left-handers compared to right-handers [33]. A Canadian study tested an association between handedness and depressive symptoms in students using self-reports of the BDI. Interestingly, left-handers had significantly higher scores than right-handers, which was only evident in males [34]. Another population-based study tested the association between handedness and external and internal problems (self-report with the Child Behavior Checklist) in over 2.000 adolescents (mean age of 14). Interestingly, they found positive associations between non-right-handedness and internal symptoms such as social and thought problems, being withdrawn and depressed. However, no association was evident between externalizing problems and handedness [35].

Furthermore, animal models of mental disorders highlight that specific symptoms of depression, such as anhedonia or behavioral despair, are significantly increased in left-lateralized animals, even demonstrating a predictive value of left-lateralized behavior for symptom severity [36–38]. However, a recent meta-analysis failed to find increased rates of non-right-handedness in patients with depression [39]. Similar results have been reported for bipolar disorders [40]. However, the meta-analysis on depression and handedness did not control for symptom severity or the kind of symptoms experienced. Notably, the studies mentioned above used self-scores of depressive symptoms. Thus, it may be possible that the pooling of sub-groups conducted in the meta-analysis diluted potential symptom-based associations found by others. Consequently, more studies analyzing an association between handedness and different symptoms of depression are needed.

In patients with schizophrenia, individuals who experience symptoms affecting the language system demonstrate a greater reduction of leftward language lateralization [41, 42]. Atypical language lateralization is also reflected in brain structure [43]. Here, symptom severity is again crucial for brain regions such as the planum temporale where the amount of decrease of leftward asymmetry correlated with symptom severity in patients [32]. The severity of positive psychotic symptoms such as delusion and hallucination has been associated with altered leftward lateralization in white matter integrity as well [44]. Consequently, it may be interesting to analyze the association between handedness and different symptoms of schizophrenia. Nevertheless, the association between diagnosis, symptom severity, and lateralization still needs to be disentangled further to identify the leading mechanism.

The present study aims to clarify whether non-specific, diagnosis-specific, or symptom-specific factors drive hemispheric asymmetries in common mental disorders. To this end, hand preference was determined in a large clinical sample from Germany.

## Methods

### Sample description

Data from the bi-center, longitudinal study cohort DFG FOR2107 were re-analyzed. This cohort study examines healthy individuals from the general population and patients with major depressive disorder (MDD), bipolar disorder (BD), schizophrenia, and schizoaffective disorder [45]. Ethics approval was obtained from the ethics committees of the Medical Schools of the Universities of Marburg (approval identifier Study 07/2014) and Münster, respectively, in accordance with the Declaration of Helsinki. All subjects volunteered to participate in the study and provided written informed consent. Baseline testing and trait data assessment were conducted in both cities between 2014 and 2018. A total of 1213 patients and 994 healthy controls are included in this study. In detail, data were available for 358 healthy men and 636 healthy women, 911 patients (322 men, 589 women) diagnosed with MDD, 154 patients (71 men, 83 women) diagnosed with BD, 52 patients (22 men, 30 women) diagnosed with schizoaffective disorder, and 96 patients (55 men, 41 women) diagnosed with schizophrenia.

### Handedness phenotyping

Handedness can be assessed with specific questionnaires such as the Edinburgh Handedness Inventory (EHI) [46]. Based on an individual’s distribution of right- and left-hand preferences for particular tasks, the experimenter can determine a laterality quotient (LQ) that indicates both direction and strength of the participant’s preference [47]. Hand preference in the study cohort DFG FOR2107 was determined in all participants with the EHI and the LQ was calculated according to the formula LQ = ((R-L) / (R + L)) × 100, with R as the number of right-hand preferences and L specifying the number of left-hand preferences. Values range between − 100 and 100, with positive values indicating a right-sided preference and negative values indicating a left-sided preference. In addition, a three-category system was used to distinguish between left-handers (LH), mixed-handers (MH), and right-handers (RH) based on LQ scores (LQ: -100 to -60: left-handed; LQ: -60 to 60 mixed-handed; LQ: 60 to 100: right-handed) [48].

### Diagnostic criteria and symptom severity

Psychopathology and symptom severity were assessed with self-report questionnaires and observer-rated inventories. The primary diagnosis was determined with the Structured Clinical Interview (SCID) for DSM-IV Axis I [49]. General symptom distress was assessed with the Global Severity Index of the Symptom Checklist-90-Revised (SCL-90-R), which queries psychological distress on nine primary symptom dimensions: somatization, obsessive-compulsive symptoms, interpersonal sensitivity, depression, anxiety, hostility, phobic anxiety, paranoid ideation, psychoticism. Additional items are also entered into the sum score (i.e., questions regarding poor appetite, difficulty falling asleep, the thought of death and dying, and feeling guilty) [50]. Global and social functioning was determined using the Global Assessment of Functioning (GAF) scale [49]. Childhood trauma (emotional, physical, or sexual abuse and emotional or physical neglect) was assessed with the Childhood Trauma Questionnaire (CTQ) [51]. Psychosocial stress was assessed with the Perceived Stress Scale (PSS) [52]. Symptoms of altered mood and anhedonia were evaluated with the Snaith-Hamilton-Pleasure Scale (SHAPS) [53]. Symptoms of depression were assessed with the Beck Depression Inventory (BDI) [54, 55] and with the Structured Interview Guide for the Hamilton Depression Rating Scale (SIGH; Hamilton Rating Scale for Depression (HAMD)) [56, 57]. Symptoms of mania were assessed with the Young Mania Rating Scale (YMRS) [58]. Symptoms of anxiety were assessed with the State-Trait Anxiety Inventory (STAIS, STAIT) [59] and the Hamilton Anxiety Scale (HAMA) [60]. Symptoms of schizophrenia were assessed with the Scale for the Assessment of Positive Symptoms (SAPS), which measures positive formal thought disorder, bizarre behavior, hallucinations, and delusions [61], and the Scale for the Assessment of Negative Symptoms (SANS), which measures apathy, affect flattening, alogia, anhedonia, and attention [62].

### Statistical analyses

Different statistical analyses were conducted to clarify whether non-specific, diagnosis-specific, or symptom-specific factors drive hemispheric asymmetries measured as handedness. First, a general difference in LQ and handedness between patients and controls was investigated using an independent samples *t*-test to investigate non-specific associations. Differences in the relative frequency of left-, mixed- and right-handedness were analyzed with Chi-Square tests. Second, independent samples t-tests were also used to investigate diagnosis-specific associations, testing for differences between the control group and distinct patient groups (MDD, BD, schizoaffective disorder, and schizophrenia). Third, to disentangle symptom-specific associations, the symptom scores from the questionnaires and inventories were correlated with the LQ, first across all groups, then within distinct patient groups, and the control group. Consequently, Pearson’s r was obtained for correlations and effect sizes were characterized as small (r = +/- 0.1 - +/- 0.3), medium (r = +/−0.3 - +/- 0.5), and large (r ≥ +/- 0.5). Furthermore, differences in handedness and symptom scores were tested using ANOVA, with handedness (LH, RH, MH) as independent factor and symptom scores as dependent variables. Again, these analyses were run across all groups and then within distinct patient and control groups. If the homogeneity of variance was not given for all measures, the Kruskal-Wallis-Test was used with handedness (LH, RH, MH) set as fixed factor and symptom scores as dependent variables. The statistical analyses and result interpretation will focus on the reported effect sizes, as suggested by Tomczak and Tomczak (2014). In line with this, effect sizes derived from the Kruskal-Wallis-Test are provided as ε^2^ (the upper and lower bounds given for Pearson’s r according to Cohen (1988)). Accordingly, effect sizes were characterized as small (ε^2^ = +/- 0.01 - +/- 0.09), medium (ε^2^ = +/−0.09 - +/- 0.25), and large (ε2 ≥ +/- 0.25). Nevertheless, p-values will be reported for the sake of completeness and comparability with other studies. Correlation coefficients were not corrected for sex and age to ensure comparability with previous clinical laterality studies. All statistical analyses were conducted using JASP [63] and SPSS (IBM Corp. Released 2020. IBM SPSS Statistics for Windows, Version 27.0. Armonk, NY: IBM Corp).

## Results

### Non-specific associations between handedness and psychopathology

**Fig. 1:**
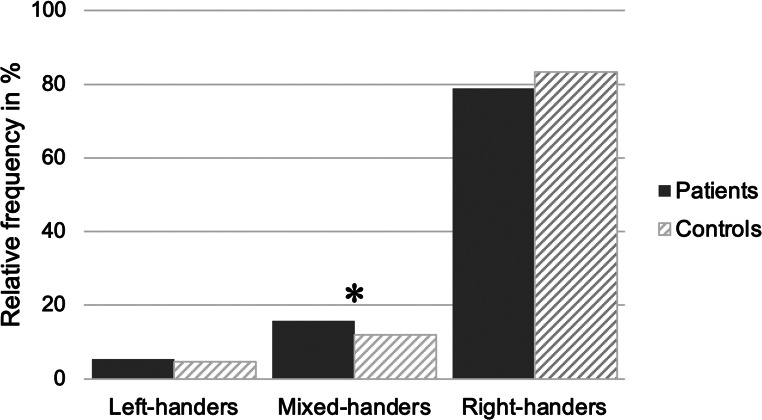
Relative frequency of left-, mixed- and right-handers in patients and healthy controls. The percentage of left-, mixed- and right-handers is shown in patients (dark grey) and healthy controls (striped grey). * p < 0.05

To assess the relationship between handedness and general psychopathology independent of a specific diagnosis, an independent samples t-test with group (controls, *n* = 994, and patients independent of diagnosis, *n* = 1213) as the pseudo-independent variable and the LQ as the dependent variable was calculated. Overall, patients (M = 71.00, SD = 50.13) showed a lower LQ than controls (M = 75.62; SD = 46.06), but the effect size was small (t_(2205)_ = 2.25; *p* < 0.05, Cohen’s d: 0.10). Moreover, the relative frequency of LH, MH, and RH were compared between patients and controls using Chi-Square tests. Overall, the effect reached significance (Chi^2^ = 7.28; *p* < 0.05), with patients showing a higher percentage of mixed-handedness (LH: 5.4%, MH: 15.7%, RH: 78.9%) than controls (LH: 4.7%, MH: 12%, RH: 83.3%) (Fig. 1).

### Diagnosis-specific associations between handedness and psychopathology

To assess the relationship between handedness and specific diagnoses, the mean LQ was compared between the control group and distinct patient groups (MDD, BD, schizoaffective disorder, and schizophrenia) through independent-sample t-tests. Interestingly, when comparing mean LQ between patients diagnosed with MDD and controls, LQ values were lower in patients (Table 1). None of the effects survived Bonferroni correction (corrected significance threshold: *p* < 0.01).

**Table 1:** LQ in different patient groups. Mean LQ per group and p-value and Cohen’s d for comparison between patient and control groups

Group	Mean LQ	Standard error	*P* comparison control	Cohen’s d comparison control
Controls	75.62	1.52	-	-
Major depressive disorder	71.03	1.59	0.04*	0.10
Bipolar disorder	72.32	3.86	0.41	0.07
Schizoaffective disorder	67.80	6.64	0.23	0.17
Schizophrenia	70.31	4.89	0.28	0.12

Effect sizes were characterized as small (d = +/- 0.2 - +/- 0.5), medium (d = +/−0.5 - +/- 0.8), and large (d = +/- 0.8 or larger). * *p < 0.05 (uncorrected)*

**Fig. 2:**
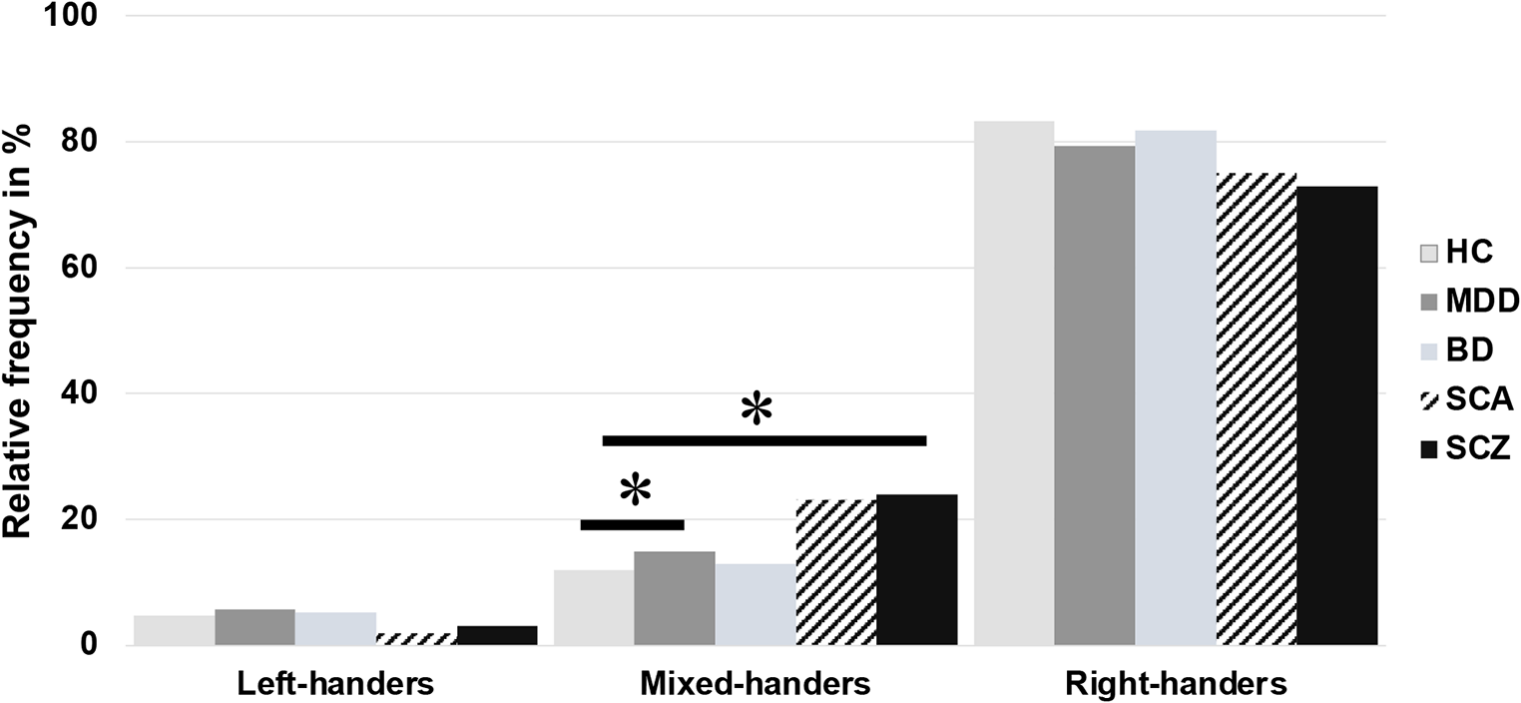
Proportion of left-, mixed-, and right-handedness in healthy controls and distinct patient groups. HC: Healthy controls; MDD: Major depressive disorder; BD: Bipolar disorder; SCA: Schizoaffective disorder; SCZ: Schizophrenia, * p < 0.05 (uncorrected)

After that, proportions of left-, right-, and mixed-handedness were analyzed in the patient and control groups using Chi-Square tests (Fig. 2).

When comparing the proportions of handedness in controls (LH: 4.7%, MH: 12%, RH: 83.3%) to MDD patients (LH: 5.8%, MH: 14.9%, RH: 79.3%), the MDD patient group included more mix-handers than the control group (*p* = 0.02). Patients with BD did not differ in prevalence (LH: 5.2%, MH: 13%, RH: 81.8%) from controls (*p* = 0.65), similar to patients with schizoaffective disorder (LH: 1.9%, MH: 23.1%, RH: 75%) compared to controls (*p* = 0.17). Differences in the percentage of mixed-handers were evident when comparing patients with schizophrenia (LH: 3.1%, MH: 24.0%, RH: 72.9%) to the control group (*p* = 0.02). The overall percentage of mixed-handers in patients with schizophrenia was even higher than in MDD. None of the effects survived Bonferroni correction (corrected significance threshold: *p* < 0.01).

### Handedness and familial predisposition

Additionally, potential effects of familial predisposition defined as having at least one first-degree relative diagnosed with a psychiatric disorder (Yes/No question) on handedness were analyzed. To this end, the frequency of left-, right-, and mixed-handers with familial predisposition were analyzed in the patient and control groups using Chi-Square tests (Table 2). There were no significant differences between the frequency of handedness type dependent on familiar predisposition in any group. A difference was prevalent in MDD patients with fewer left-handers with no first-degree relatives (Bonferroni corrected significance threshold: *p* < 0.025).

**Table 2:** Frequency of left-, mixed- or right-handers dependent on familiar predisposition in different patient groups

	First-degree relative	Left-handers %	Mixed-handers %	Right-handers %	*p*-value (uncorrected)
All	Yes	2.6	6.7	41.2	> 0.05
	No	2.5	7.3	39.7	
Controls	Yes	1.7	4.2	28.3	> 0.05
	No	3.1	7.9	54.8	
Major depressive disorder	Yes	3.8	8.4	55.4	0.005*
	No	1.9	6.9	23.7	
Bipolar disorder	Yes	3.6	9.3	50.0	> 0.05
	No	1.4	3.6	32.1	
Schizoaffective disorder	Yes	2.6	5.3	50.0	> 0.05
	No	0	13.2	28.9	
Schizophrenia	Yes	0	15.0	32.5	> 0.05
	No	3.8	8.8	40.0	

Familiar predisposition is defined as having at least one first-degree relative with a psychiatric diagnosis (Yes/No). * p < 0.05 (uncorrected)

### Symptom-specific associations between handedness and cross-sectional clinical severity measures independent of diagnosis

Cross-sectional clinical severity measures were correlated with the LQ to investigate symptom-specific associations in the whole sample. Consequently, Pearson’s r will be reported for correlations. Only correlations reaching a medium effect size or larger will be explicitly named; all other information is provided in the tables.

Analyzing the whole sample revealed no correlation with a small, medium, or large effect size (r ≥ +/- 0.1) (Table 3). All correlation coefficients were between *r* = -0.1 and *r* = 0.1, indicating no substantial relation between symptom severity and hand preference. None of the effects survived Bonferroni correction (corrected significance threshold: *p* < 0.0013).

**Table 3:** Correlation coefficient r for LQ and current/cross sectional clinical severity measures in controls (*n* = 994) and patients (*n* = 1213) independent of diagnosis

Measure	Scoring	Pearson’s *r*	*p*-value
GAF	Total score	0.052*	0.017
PSS sum score	Total score	-0.039	0.065
STAIS sum score	Total score	-0.064**	0.003
STAIT sum score	Total score	-0.053*	0.015
SIGH sum score	Total score	-0.058**	0.008
YMRS sum score	Total score	-0.042*	0.047
BDI sum score	Total score	-0.064**	0.003
SHAPS sum score	Total score	-0.048*	0.027
HAMA sum score	Total score	-0.048*	0.026
HAMD	Total score (21-items)	-0.054*	0.011
CTQ	Total score	-0.033	0.128
	Emotional abuse	-0.035	0.103
	Emotional neglect	-0.027	0.209
	Physical abuse	-0.022	0.307
	Physical neglect	-0.017	0.426
	Sexual abuse	-0.028	0.191
SANS	Total score	-0.042*	0.049
	Abulia, apathy	-0.048*	0.024
	Affect flattening	-0.030	0.166
	Alogia	-0.037	0.086
	Anhedonia	-0.024	0.258
	Attention	-0.028	0.186
SAPS	Total sum	-0.043*	0.046
	Positive formal thought disorder	-0.048*	0.026
	Bizarre behavior	0.006	0.781
	Hallucinations	-0.044*	0.041
	Delusions	-0.021	0.337
SCL90R	Global severity index	-0.051*	0.017
	Anxiety	-0.036	0.092
	Hostility	-0.035	0.101
	Depression	-0.043*	0.047
	Paranoid ideation	-0.056	0.009
	Phobic anxiety	-0.027	0.209
	Psychoticism	-0.055*	0.011
	Somatization	-0.047*	0.028
	Interpersonal sensitivity	-0.057**	0.007
	Additional items	-0.057**	0.007
	Obsessive-compulsive	-0.033	0.122

A negative r indicates higher symptom severity / higher values for a parameter with lower LQ (i.e., increasing tendency towards left-handedness). A positive r indicates higher symptom severity with higher LQ (i.e., increasing tendency towards right-handedness). This applies to all measures except for the GAF where lower scores reflect lower global functioning. Effect sizes were characterized as small (r = +/- 0.1 - +/- 0.3), medium (r = +/−0.3 - +/- 0.5), and large (r = +/- 0.5 or larger). * p < 0.05 (uncorrected), ** p < 0.01

### Symptom-specific associations between handedness and cross-sectional clinical severity measures dependent on diagnosis

**Fig. 3:**
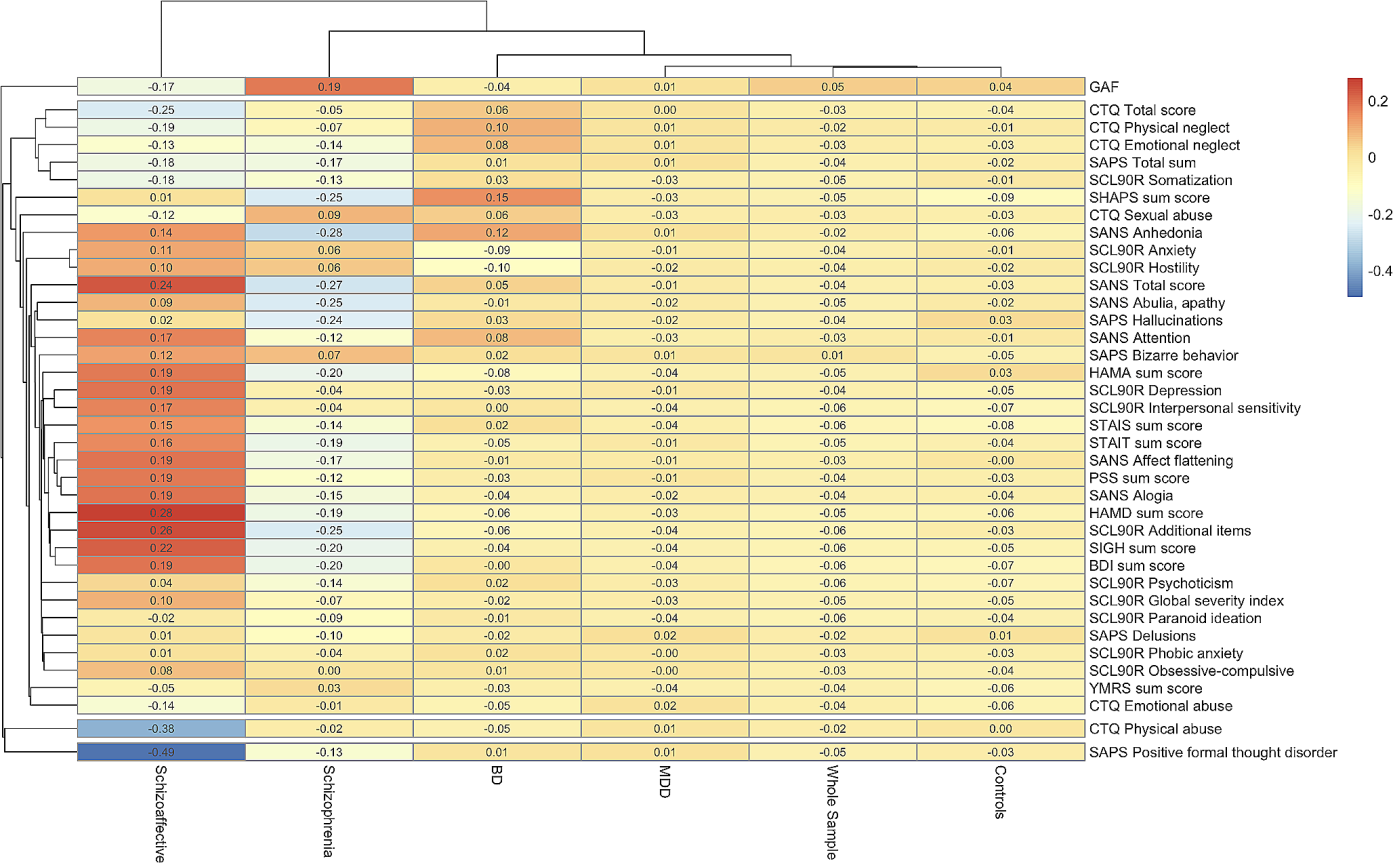
Heatmap of Pearson’s r for symptom-specific associations between handedness and cross-sectional clinical severity measures for all groups. A negative r (blue colors) indicates higher symptom severity / higher values for a parameter with lower LQ (i.e., increasing tendency towards left-handedness). A positive r (red colors) indicates higher symptom severity with higher LQ (i.e., increasing tendency towards right-handedness). R-values around zero (yellow colors) indicate weak associations between LQ and symptom severity

To analyze the association between left- and right-handers and cross-sectional clinical severity measures dependent on diagnosis, symptom scores were correlated with the LQ separately, first only for controls, then for patients with MDD, patients with BD, patients with schizoaffective disorder, and patients with schizophrenia. Consequently, Pearson’s r will be reported for correlations. Only correlations reaching a medium effect size will be explicitly named, all other information is provided in the tables. The effect sizes (Pearson’s r) of all correlations for all groups are visualized in Fig. 3.

From a total of 38 correlations, none even reached a small effect size (r ≥ +/- 0.1) when analyzing only controls (Table 4), indicating no association between sub-clinical symptom severity and LQ. None of the effects survived Bonferroni correction (corrected significance threshold: *p* < 0.0013).

**Table 4:** Correlation coefficient r for LQ and current/cross sectional clinical severity measures in clinically healthy participants (*n* = 994)

Measure	Scoring	Pearson’s *r*	*p*-value
GAF	Total score	0.042	0.189
PSS sum score	Total score	-0.030	0.340
STAIS sum score	Total score	-0.080**	0.012
STAIT sum score	Total score	-0.043	0.179
SIGH sum score	Total score	-0.054	0.095
YMRS sum score	Total score	-0.063*	0.046
BDI sum score	Total score	-0.074*	0.020
SHAPS sum score	Total score	-0.085**	0.008
HAMA sum score	Total score	0.026	0.413
HAMD	Total score (21-items)	-0.056	0.077
CTQ	Total score	-0.038	0.229
	Emotional abuse	-0.055	0.081
	Emotional neglect	-0.030	0.351
	Physical abuse	0.002	0.953
	Physical neglect	-0.013	0.675
	Sexual abuse	-0.029	0.362
SANS	Total score	-0.033	0.306
	Abulia, apathy	-0.019	0.540
	Affect flattening	-0.002	0.961
	Alogia	-0.035	0.267
	Anhedonia	-0.058	0.068
	Attention	-0.012	0.715
SAPS	Total sum	-0.021	0.510
	Positive formal thought disorder	-0.027	0.390
	Bizarre behavior	-0.046	0.149
	Hallucinations	0.031	0.325
	Delusions	0.012	0.697
SCL90R	Global severity index	-0.049	0.120
	Anxiety	-0.010	0.742
	Hostility	-0.018	0.568
	Depression	-0.052	0.100
	Paranoid ideation	-0.044	0.162
	Phobic anxiety	-0.030	0.341
	Psychoticism	-0.065*	0.041
	Somatization	-0.006	0.854
	Interpersonal sensitivity	-0.073*	0.021
	Additional items	-0.026	0.421
	Obsessive-compulsive	-0.037	0.246

A negative r indicates higher symptom severity / higher values for a parameter with lower LQ (i.e., increasing tendency towards left-handedness). A positive r indicates higher symptom severity with higher LQ (i.e., increasing tendency towards right-handedness). This applies to all measures except for the GAF where lower scores reflect lower global functioning. Effect sizes were characterized as small (r = +/- 0.1 - +/- 0.3), medium (r = +/−0.3 - +/- 0.5), and large (r = +/- 0.5 or larger). * p < 0.05 (uncorrected), ** p < 0.01

Similarly, none out of 38 correlations reached a small effect size when analyzing patients with MDD, indicating no association between symptom severity and hand preference (Table 5). None of the effects survived Bonferroni correction (corrected significance threshold: *p* < 0.0013).

**Table 5:** Correlation coefficient r for LQ and current/cross sectional clinical severity measures in patients diagnosed with MDD (*n* = 911)

Measure	Scoring	Pearson’s *r*	*p*-value
GAF	Total score	0.014	0.678
PSS sum score	Total score	-0.005	0.873
STAIS sum score	Total score	-0.035	0.287
STAIT sum score	Total score	-0.011	0.731
SIGH sum score	Total score	-0.045	0.191
YMRS sum score	Total score	-0.041	0.218
BDI sum score	Total score	-0.042	0.213
SHAPS sum score	Total score	-0.026	0.431
HAMA sum score	Total score	-0.036	0.276
HAMD	Total score (21-items)	-0.030	0.371
CTQ	Total score	0.003	0.939
	Emotional abuse	0.016	0.635
	Emotional neglect	0.010	0.764
	Physical abuse	0.007	0.837
	Physical neglect	0.006	0.848
	Sexual abuse	-0.028	0.396
SANS	Total score	-0.014	0.685
	Abulia, apathy	-0.019	0.563
	Affect flattening	-0.014	0.674
	Alogia	-0.021	0.526
	Anhedonia	0.015	0.653
	Attention	-0.030	0.371
SAPS	Total sum	0.014	0.667
	Positive formal thought disorder	0.014	0.679
	Bizarre behavior	0.006	0.849
	Hallucinations	-0.019	0.559
	Delusions	0.023	0.486
SCL90R	Global severity index	-0.025	0.451
	Anxiety	-0.015	0.651
	Hostility	-0.016	0.630
	Depression	-0.010	0.762
	Paranoid ideation	-0.036	0.284
	Phobic anxiety	-0.001	0.972
	Psychoticism	-0.028	0.403
	Somatization	-0.033	0.327
	Interpersonal sensitivity	-0.041	0.223
	Additional items	-0.042	0.202
	Obsessive-compulsive	-3.225 × 10^− 4^	0.992

A negative r indicates higher symptom severity / higher values for a parameter with lower LQ (i.e., increasing tendency towards left-handedness). A positive r indicates higher symptom severity with higher LQ (i.e., increasing tendency towards right-handedness). This applies to all measures except for the GAF where lower scores reflect lower global functioning. Effect sizes were characterized as small (r = +/- 0.1 - +/- 0.3), medium (r = +/−0.3 - +/- 0.5), and large (r = +/- 0.5 or larger)

However, of the 38 correlations analyzed for patients with BD, three showed small effect sizes, with higher scores in hostility linked to left-handedness and higher SAPS and SANS scores for anhedonia linked to right-handedness (Table 6). None of the effects survived Bonferroni correction (corrected significance threshold: *p* < 0.0013).

**Table 6:** Correlation coefficient r for LQ and current/cross sectional clinical severity measures in patients diagnosed with BD (*n* = 154)

Measure	Scoring	Pearson’s *r*	*p*-value
GAF	Total score	-0.035	0.676
PSS sum score	Total score	-0.026	0.745
STAIS sum score	Total score	0.023	0.778
STAIT sum score	Total score	-0.050	0.542
SIGH sum score	Total score	-0.042	0.605
YMRS sum score	Total score	-0.032	0.693
BDI sum score	Total score	-0.003	0.973
SHAPS sum score	Total score	0.153	0.060
HAMA sum score	Total score	-0.076	0.349
HAMD	Total score (21-items)	-0.064	0.433
CTQ	Total score	0.063	0.438
	Emotional abuse	-0.054	0.508
	Emotional neglect	0.081	0.317
	Physical abuse	-0.050	0.536
	Physical neglect	0.096	0.236
	Sexual abuse	0.056	0.489
SANS	Total score	0.046	0.574
	Abulia, apathy	-0.009	0.915
	Affect flattening	-0.009	0.914
	Alogia	-0.039	0.636
	Anhedonia	0.116	0.154
	Attention	0.083	0.309
SAPS	Total sum	0.012	0.883
	Positive formal thought disorder	0.008	0.920
	Bizarre behavior	0.022	0.788
	Hallucinations	0.030	0.712
	Delusions	-0.018	0.824
SCL90R	Global severity index	-0.023	0.774
	Anxiety	-0.091	0.263
	Hostility	-0.105	0.196
	Depression	-0.026	0.752
	Paranoid ideation	-0.009	0.910
	Phobic anxiety	0.016	0.842
	Psychoticism	0.023	0.775
	Somatization	0.025	0.760
	Interpersonal sensitivity	0.002	0.981
	Additional items	-0.063	0.443
	Obsessive-compulsive	0.009	0.914

A negative r indicates higher symptom severity / higher values for a parameter with lower LQ (i.e., increasing tendency towards left-handedness). A positive r indicates higher symptom severity with higher LQ (i.e., increasing tendency towards right-handedness). This applies to all measures except for the GAF where lower scores reflect lower global functioning. Effect sizes were characterized as small (r = +/- 0.1 - +/- 0.3), medium (r = +/−0.3 - +/- 0.5), and large (r = +/- 0.5 or larger)

Interestingly, for patients with schizoaffective disorder, out of the 38 correlations, 27 showed a small effect size (r > +/- 0.1) and two medium effect sizes (r > +/−0.3). Eight of the 27 correlations with small effect sizes indicated increased scores in left-handers, while the remaining indicated higher symptom severity in right-handers. Medium effect sizes were reached for CTQ physical abuse scores, as well as for the SAPS formal thought disorder scores, with left-handers showing higher symptom severity compared to right-handers. The significant effect found for the SAPS survived Bonferroni correction (*p* < 0.001). All results are shown in Table 7.

**Table 7:** Correlation coefficient r for LQ and current/cross sectional clinical severity measures in patients diagnosed with schizoaffective disorder (*n* = 52)

Measure	Scoring	Pearson’s *r*	*p*-value
GAF	Total score	-0.174	0.238
PSS sum score	Total score	0.188	0.187
STAIS sum score	Total score	0.146	0.300
STAIT sum score	Total score	0.161	0.263
SIGH sum score	Total score	0.221	0.123
YMRS sum score	Total score	-0.052	0.715
BDI sum score	Total score	0.192	0.186
SHAPS sum score	Total score	0.012	0.932
HAMA sum score	Total score	0.188	0.181
HAMD sum score	Total score (21-items)	0.281*	0.043
CTQ	Total score	-0.248	0.079
	Emotional abuse	-0.139	0.332
	Emotional neglect	-0.127	0.373
	Physical abuse	-0.377**	0.006
	Physical neglect	-0.189	0.179
	Sexual abuse	-0.124	0.385
SANS	Total score	0.237	0.094
	Abulia, apathy	0.089	0.532
	Affect flattening	0.192	0.178
	Alogia	0.190	0.177
	Anhedonia	0.138	0.330
	Attention	0.167	0.236
SAPS	Total sum	-0.181	0.205
	Positive formal thought disorder	-0.492***#	< 0.001
	Bizarre behavior	0.119	0.402
	Hallucinations	0.018	0.902
	Delusions	0.010	0.944
SCL90R	Global severity index	0.096	0.503
	Anxiety	0.113	0.430
	Hostility	0.104	0.468
	Depression	0.194	0.172
	Paranoid ideation	-0.020	0.890
	Phobic anxiety	0.012	0.936
	Psychoticism	0.039	0.786
	Somatization	-0.184	0.196
	Interpersonal sensitivity	0.168	0.240
	Additional items	0.255	0.071
	Obsessive-compulsive	0.080	0.576

A negative r indicates higher symptom severity / higher values for a parameter with lower LQ (i.e., increasing tendency towards left-handedness). A positive r indicates higher symptom severity with higher LQ (i.e., increasing tendency towards right-handedness). This applies to all measures except for the GAF where lower scores reflect lower global functioning. Effect sizes were characterized as small (r = +/- 0.1 - +/- 0.3), medium (r = +/−0.3 - +/- 0.5), and large (r = +/- 0.5 or larger). * p < 0.05 (uncorrected), ** p < 0.01, # p < 0.0013 (Bonferroni corrected)

Similarly, correlation analyses for LQ and severity measures only for patients with schizophrenia yielded correlations for 21 measures that showed a small effect size. Of these 21 correlations with small effect sizes, 20 indicated higher symptom severity in left- than right-handers. Right-handers, accordingly, showed higher GAF scores than left-handers. Detailed results are shown in Table 8. None of the effects survived Bonferroni correction (corrected significance threshold: *p* < 0.0013).

**Table 8:** Correlation coefficient r for LQ and current/cross sectional clinical severity measures in patients diagnosed with schizophrenia (*n* = 96)

Measure	Scoring	Pearson’s *r*	*p*-value
GAF	Total score	0.188	0.077
PSS sum score	Total score	-0.120	0.249
STAIS sum score	Total score	-0.138	0.186
STAIT sum score	Total score	-0.194	0.063
SIGH sum score	Total score	-0.203*	0.049
YMRS sum score	Total score	0.026	0.804
BDI sum score	Total score	-0.196	0.060
SHAPS sum score	Total score	-0.246*	0.016
HAMA sum score	Total score	-0.201	0.051
HAMD sum score	Total score (21-items)	-0.189	0.066
CTQ	Total score	-0.052	0.616
	Emotional abuse	-0.008	0.937
	Emotional neglect	-0.145	0.160
	Physical abuse	-0.024	0.818
	Physical neglect	-0.070	0.500
	Sexual abuse	0.091	0.383
SANS	Total score	-0.266*	0.010
	Abulia, apathy	-0.248*	0.016
	Affect flattening	-0.170	0.100
	Alogia	-0.153	0.141
	Anhedonia	-0.285**	0.005
	Attention	-0.121	0.244
SAPS	Total sum	-0.173	0.095
	Positive formal thought disorder	-0.134	0.195
	Bizarre behavior	0.073	0.483
	Hallucinations	-0.237*	0.021
	Delusions	-0.096	0.359
SCL90R	Global severity index	-0.068	0.520
	Anxiety	0.056	0.596
	Hostility	0.062	0.552
	Depression	-0.042	0.690
	Paranoid ideation	-0.094	0.369
	Phobic anxiety	-0.038	0.715
	Psychoticism	-0.143	0.171
	Somatization	-0.133	0.200
	Interpersonal sensitivity	-0.039	0.713
	Additional items	-0.246*	0.017
	Obsessive- compulsive	0.003	0.980

A negative r indicates higher symptom severity / higher values for a parameter with lower LQ (i.e., increasing tendency towards left-handedness). A positive r indicates higher symptom severity with higher LQ (i.e., increasing tendency towards right-handedness). This applies to all measures except for the GAF where lower scores reflect lower global functioning. Effect sizes were characterized as small (r = +/- 0.1 - +/- 0.3), medium (r = +/−0.3 - +/- 0.5), and large (r = +/- 0.5 or larger). * p < 0.05 (uncorrected), ** p < 0.01

## Symptom severity comparison between left-handers, right-handers, and mixed-handers

Furthermore, handedness and symptom severity differences were analyzed once in the whole patient group (4.1.) and once within the distinct patient groups (4.2). The Kruskal-Wallis-Test was used for these analyses, with handedness (LH, RH, MH) as fixed factor and symptom scores as dependent variables. Accordingly, effect sizes are given in ε^2^ and are characterized as small (ε^2^ = +/- 0.01 - +/- 0.09), medium (ε^2^ = +/−0.09 - +/- 0.25), and large (ε^2^ = +/- 0.25). None of the 24 measures reached an effect size of ε^2^ ≥ +/- 0.01. Only comparisons reaching a medium effect size will be explicitly named, all other information is given in the tables.

### Symptom severity comparison between left-handers, right-handers, and mixed-handers independent of diagnosis

In the analyses for the whole sample, none of the 24 measures reached a small effect size, with six of the effects survived Bonferroni correction (corrected significance threshold: *p* < 0.0021). (Table 9), indicating no differences in symptom severity between left-, mixed- and right-handers.

**Table 9:** Descriptive statistics and epsilon-squared estimate of effect size based on the Kruskal-Wallis-Test for sum score effects for controls (*n* = 994) and patients (*n* = 1213) independent of diagnosis

Measure (sum scores)	LH mean	MH mean	RH mean	Effect size ε^2^	*p*-value
BDI	11.68	**13.08**	10.69	0.007	< 0.001***#
CTQ	38.95	**41.41**	39.25	0.005	< 0.05*
GAF	74.86	**72.90**	76.37	0.004	< 0.05*
HAMA	8.40	**8.95**	7.57	0.004	< 0.05*
HAMD 21	6.28	**6.51**	5.45	0.006	< 0.05*
PSS	23.65	**24.68**	22.97	0.003	< 0.05*
SANS	4.93	**5.84**	4.61	0.004	< 0.05*
SAPS	0.77	**2.41**	0.96	0.008	< 0.001***#
SCL90R global severity index	0.64	**0.77**	0.62	0.006	< 0.01**
SCL90R anxiety	5.26	**6.41**	5.26	0.003	< 0.05*
SCL90R hostility	2.79	**3.73**	2.87	0.005	< 0.01**
SCL90R depression	11.97	**13.48**	11.52	0.004	< 0.05*
SCL90R paranoid ideation	3.53	**4.30**	3.27	0.005	< 0.01**
SCL90R phobic anxiety	2.14	**2.93**	2.29	0.005	< 0.01**
SCL90R psychoticism	3.76	**5.01**	3.69	0.010	< 0.001***#
SCL90R somatization	6.89	**8.84**	6.89	0.004	< 0.01**
SCL90R interpersonal sensitivity	7.56	**7.95**	6.63	0.003	< 0.05*
SCL90R additional items	4.42	**5.17**	4.07	0.009	< 0.001***#
SCL90R obsessive-compulsive	8.21	**9.99**	8.38	0.006	< 0.01**
SHAPS	**2.37**	2.30	1.97	0.004	< 0.01**
SIGH	8.35	**9.21**	7.33	0.007	< 0.001***#
STAIS	44.08	**44.49**	42.28	0.004	< 0.05*
STAIT	45	45.32	43.51	0.003	> 0.05
YMRS	1.37	**1.83**	1.23	0.008	< 0.001***#

For the means (M) the mean of the group (LH: left-handers; MH: mixed-handers; RH: right-handers) with the highest or lowest value (for GAF) is marked in bold. For interpreting effect sizes, the upper and lower bounds given for Pearson’s r according to Cohen (1988) were squared. Accordingly, effect sizes were characterized as small ε^2^ = +/- 0.01 - +/- 0.09), medium (ε^2^ = +/−0.09 - +/- 0.25) and large (ε^2^ = +/- 0.25). * p < 0.05 (uncorrected), ** p < 0.01, *** p < 0.001, # p < 0.0021 (Bonferroni corrected)

### Symptom severity comparison between left-handers, right-handers, and mixed-handers dependent on diagnosis

In the control group, out of 24 comparisons, two reached a small effect size (Table 10), indicating more severe SHAPS symptoms in left- than right-handers. None of the effects survived Bonferroni correction (corrected significance threshold: *p* < 0.0021).

**Table 10:** Descriptive statistics and epsilon-squared estimate of effect size based on the Kruskal-Wallis-Test for sum score effects for healthy controls (*n* = 994)

Measure (sum scores)	LH mean	MH mean	RH mean	Effect size ε^2^	*p*-value
BDI	4.98	5.20	3.98	0.010	< 0.01**
CTQ	33.26	33.23	32.64	0.004	> 0.05
GAF	91.1	90.5	91.31	< 0.000	> 0.05
HAMA	2.28	2.86	2.78	0.002	> 0.05
HAMD 21	1.45	1.99	1.39	0.006	> 0.05
PSS	17.02	17.6	16.16	0.002	> 0.05
SANS	0.68	0.77	0.57	0.003	> 0.05
SAPS	0.04	0.30	0.15	0.002	> 0.05
SCL90R global severity index	0.24	0.30	0.23	0.007	< 0.05*
SCL90R anxiety	1.57	1.88	1.67	0.002	> 0.05
SCL90R hostility	1.15	1.55	1.22	0.002	> 0.05
SCL90R depression	4.09	5.14	3.75	0.005	> 0.05
SCL90R paranoid ideation	1.23	1.76	1.24	0.002	> 0.05
SCL90R phobic anxiety	0.43	0.63	0.43	0.002	> 0.05
SCL90R psychoticism	0.98	1.76	1.05	0.008	< 0.05*
SCL90R somatization	3.45	3.95	3.66	0.001	> 0.05
SCL90R interpersonal sensitivity	3.21	3.15	2.5	0.002	> 0.05
SCL90R additional items	1.87	2.45	1.9	0.010	< 0.01**
SCL90R obsessive-compulsive	3.11	**4.34**	3.25	0.012	< 0.01**
SHAPS	**1.04**	0.92	0.61	0.012	< 0.01**
SIGH	1.91	2.68	1.87	0.008	< 0.05*
STAIS	37.04	36.42	34.18	0.008	< 0.05*
STAIT	34.74	34.72	33.54	0.001	> 0.05
YMRS	0.77	0.73	0.59	0.002	> 0.05

For the means (M) the mean of the group (LH: left-handers; MH: mixed-handers; RH: right-handers) with the highest or lowest value (for GAF) is marked in bold. For interpreting effect sizes, the upper and lower bounds given for Pearson’s r according to Cohen (1988) were squared. Accordingly, effect sizes were characterized as small ε^2^ = +/- 0.01 - +/- 0.09), medium (ε^2^ = +/−0.09 - +/- 0.25) and large (ε^2^ = +/- 0.25). * p < 0.05 (uncorrected), ** p < 0.01

In patients with MDD, no comparisons reached a small effect size (Table 11), indicating no differences in symptom severity between left-, mixed-, and right-handers. None of the effects survived Bonferroni correction (corrected significance threshold: *p* < 0.0021).

**Table 11:** Descriptive statistics and epsilon-squared estimate of effect size based on the Kruskal-Wallis-Test for sum score effects for patients diagnosed with MDD (*n* = 911)

Measure (sum scores)	LH mean	MH mean	RH mean	Effect size ε^2^	*p*-value
BDI	17.23	19.83	17.61	0.003	> 0.05
CTQ	43.48	46.83	45.51	0.004	> 0.05
GAF	63.83	64.50	64.63	< 0.000	> 0.05
HAMA	12.83	13.88	12.49	0.002	> 0.05
HAMD 21	9.64	10.27	9.37	0.002	> 0.05
PSS	28.67	30.44	29.84	0.002	> 0.05
SANS	7.13	8.65	7.68	0.003	> 0.05
SAPS	0.34	1.04	0.62	0.002	> 0.05
SCL90R global severity index	0.96	1.11	1.0	0.003	> 0.05
SCL90R anxiety	8.28	9.24	8.7	0.001	> 0.05
SCL90R hostility	4.08	5.55	4.69	0.005	> 0.05
SCL90R depression	18.4	20.56	19.39	0.002	> 0.05
SCL90R paranoid ideation	5.3	5.81	5.14	0.002	> 0.05
SCL90R phobic anxiety	3.43	4.14	4.01	0.002	> 0.05
SCL90R psychoticism	5.83	6.88	6.02	0.003	> 0.05
SCL90R somatization	9.6	12.5	10.19	0.005	> 0.05
SCL90R interpersonal sensitivity	11.11	11.73	10.68	0.002	> 0.05
SCL90R additional items	6.28	7.13	6.15	0.006	> 0.05
SCL90R obsessive-compulsive	12.3	14.1	13.33	0.002	> 0.05
SHAPS	3.57	3.63	3.46	< 0.000	> 0.05
SIGH	13.11	14.79	12.54	0.006	> 0.05
STAIS	50.15	51.37	50.55	< 0.000	> 0.05
STAIT	53.11	53.61	53.74	< 0.000	> 0.05
YMRS	1.58	1.62	1.37	0.007	< 0.05*

For the means (M) the mean of the group (LH: left-handers; MH: mixed-handers; RH: right-handers) with the highest or lowest value (for GAF) is marked in bold. For interpreting effect sizes, the upper and lower bounds given for Pearson’s r according to Cohen (1988) were squared. Accordingly, effect sizes were characterized as small ε^2^ = +/- 0.01 - +/- 0.09), medium (ε^2^ = +/−0.09 - +/- 0.25) and large (ε^2^ = +/- 0.25). * p < 0.05 (uncorrected)

Six out of 24 comparisons for patients with BD reached a small effect size, with four of them highlighting higher symptom scores in mixed-handers than in left- or right-handers (Table 12). None of the effects survived Bonferroni correction (corrected significance threshold: *p* < 0.0021).

**Table 12:** Descriptive statistics and epsilon-squared estimate of effect size based on the Kruskal-Wallis-Test results for sum score effects for patients diagnosed with BD (*n* = 154)

Measure (sum scores)	LH mean	MH mean	RH mean	Effect size ε^2^	*p*-value
BDI	12.63	12.8	13.45	0.001	> 0.05
CTQ	38.14	42.85	43.18	0.002	> 0.05
GAF	63.5	62.41	62.32	< 0.000	> 0.05
HAMA	12.25	9.63	9.33	0.004	> 0.05
HAMD 21	9.38	6.79	7.2	0.008	> 0.05
PSS	27.5	26.3	26.38	0.002	> 0.05
SANS	5.25	3.79	6.02	0.009	> 0.05
SAPS	2.13	2.74	2.38	0.003	> 0.0
SCL90R global severity index	0.79	0.95	0.78	0.004	> 0.05
SCL90R anxiety	7.0	**10.42**	6.33	0.028	> 0.05
SCL90R hostility	4.63	**5.26**	3.11	0.019	> 0.05
SCL90R depression	14.63	15.11	14.52	< 0.000	> 0.05
SCL90R paranoid ideation	4.5	4.68	3.91	0.003	> 0.05
SCL90R phobic anxiety	3.25	3.42	2.92	< 0.000	> 0.05
SCL90R psychoticism	3.86	5.37	4.74	0.005	> 0.05
SCL90R somatization	5.63	11.26	8.25	0.007	> 0.05
SCL90R interpersonal sensitivity	**10.13**	7.11	7.94	0.011	> 0.05
SCL90R additional items	6.25	**7.21**	5.44	0.010	> 0.05
SCL90R obsessive-compulsive	9.5	13.74	11.29	0.008	> 0.05
SHAPS	1.25	1.42	**2.53**	0.015	> 0.05
SIGH	12.88	9.42	10.5	0.005	> 0.05
STAIS	44.63	44.79	45.68	< 0.000	> 0.05
STAIT	49.38	46.45	47.33	0.004	> 0.05
YMRS	3.25	**7.89**	3.56	0.050	< 0.05*

For the means (M) the mean of the group (LH: left-handers; MH: mixed-handers; RH: right-handers) with the highest or lowest value (for GAF) is marked in bold. For interpreting effect sizes, the upper and lower bounds given for Pearson’s r according to Cohen (1988) were squared. Accordingly, effect sizes were characterized as small ε^2^ = +/- 0.01 - +/- 0.09), medium (ε^2^ = +/−0.09 - +/- 0.25) and large (ε^2^ = +/- 0.25). * p < 0.05 (uncorrected)

In patients diagnosed with schizoaffective disorder, out of the 24 comparisons, 20 reached a small effect size, with 12 indicating higher symptom severity in right-handers, four indicating higher symptom severity in left-handers, and four showing higher symptom severity in mixed-handers (Table 13). Moreover, two comparisons reached a medium effect size, indicating higher symptom severity in right-handers. None of the effects survived Bonferroni correction (corrected significance threshold: *p* < 0.0021). However, it is important to keep in mind that this group only included one left-handed individual.

**Table 13:** Descriptive statistics and epsilon-squared estimate of effect size based on the Kruskal-Wallis-Test for sum score effects for patients diagnosed with schizoaffective disorder (*n* = 52)

Measure (sum scores)	LH mean	MH mean	RH mean	Effect size ε^2^	*p*-value
BDI	0	11.75	**15.42**	0.072	> 0.05
CTQ	**60.0**	53.82	45.08	0.073	> 0.05
GAF	71.0	57.58	**54.0**	0.065	> 0.05
HAMA	4.0	8.67	**11.31**	0.040	> 0.05
HAMD 21	4.0	4.83	**10.18**	0.124	< 0.05*
PSS	15.0	25.58	**28.68**	0.049	> 0.05
SANS	0	7.42	**12.16**	0.104	> 0.05
SAPS	**21.0**	6.17	6.45	0.053	> 0.05
SCL90R global severity index	**0.32**	0.99	0.99	0.023	> 0.05
SCL90R anxiety	2.0	9.92	**10.29**	0.033	> 0.05
SCL90R hostility	1.0	3.0	3.29	0.010	> 0.05
SCL90R depression	1.0	14.67	**17.58**	0.060	> 0.05
SCL90R paranoid ideation	3.0	6.25	5.69	0.009	> 0.05
SCL90R phobic anxiety	0.0	**6.25**	4.76	0.046	> 0.05
SCL90R psychoticism	2.0	**8.67**	7.68	0.026	> 0.05
SCL90R somatization	13.0	13.33	8.66	0.096	> 0.05
SCL90R interpersonal sensitivity	1.0	9.42	**11.74**	0.065	> 0.05
SCL90R additional items	0	5.0	**6.82**	0.092	> 0.05
SCL90R obsessive-compulsive	6.0	**12.0**	**12.03**	0.018	> 0.05
SHAPS	0	**1.92**	1.67	0.023	> 0.05
SIGH	10.0	7.42	**13.57**	0.071	> 0.05
STAIS	21.0	43.25	**46.33**	0.059	> 0.05
STAIT	30.0	46.82	**51.05**	0.071	> 0.05
YMRS	**6.0**	2.25	2.77	0.036	> 0.05

For the means (M) the mean of the group (LH: left-handers; MH: mixed-handers; RH: right-handers) with the highest or lowest value (for GAF) is marked in bold. For interpreting effect sizes, the upper and lower bounds given for Pearson’s r according to Cohen (1988) were squared. Accordingly, effect sizes were characterized as small ε^2^ = +/- 0.01 - +/- 0.09), medium (ε^2^ = +/−0.09 - +/- 0.25, light green) and large (ε^2^ = +/- 0.25). * p < 0.05 (uncorrected)

In patients with schizophrenia, 19 out of 24 comparisons reached a small effect size, and only one comparison reached a medium effect size, indicating higher SAPS scores for mix-handers than left- or right-handers. Out of the 19 comparisons with small effect sizes, 15 indicated higher scores in left-handers, and four indicated higher scores in mixed-handers. None of the effects survived Bonferroni correction (corrected significance threshold: *p* < 0.0021) (see Table 14). Interestingly, left-handers also had the lowest scores in the GAF.

**Table 14:** Descriptive statistics and epsilon-squared estimate of effect size based on the Kruskal-Wallis-Test for sum score effects for patients diagnosed with schizophrenia (*n* = 96)

Measure (sum scores)	LH mean	MH mean	RH mean	Effect size ε^2^	*p*-value
BDI	**20.0**	14.83	12.6	0.054	> 0.05
CTQ	44.67	45.52	43.22	0.009	> 0.05
GAF	**43.33**	45.0	55.47	0.072	< 0.05*
HAMA	**17.33**	11.17	8.54	0.075	< 0.05*
HAMD 21	**15.0**	7.87	7.49	0.050	> 0.05
PSS	**33.0**	25.0	23.37	0.057	> 0.05
SANS	**34.0**	16.26	14.22	0.035	> 0.05
SAPS	9.33	**19.04**	8.66	0.142	< 0.01**
SCL90R global severity index	**1.02**	0.92	0.84	0.022	> 0.05
SCL90R anxiety	6.0	8.14	7.85	0.001	> 0.05
SCL90R hostility	**1.67**	3.41	3.19	0.018	> 0.05
SCL90R depression	18.67	13.09	14.19	0.009	> 0.05
SCL90R paranoid ideation	5.67	**7.36**	5.75	0.048	> 0.05
SCL90R phobic anxiety	4.0	**5.73**	4.35	0.020	> 0.05
SCL90R psychoticism	**11.0**	8.73	7.24	0.038	> 0.05
SCL90R somatization	**14.33**	8.35	8.0	0.045	> 0.05
SCL90R interpersonal sensitivity	8.33	**10.64**	8.88	0.025	> 0.05
SCL90R additional items	**8.0**	6.14	4.47	0.087	< 0.05*
SCL90R obsessive-compulsive	13.0	11.0	10.99	0.005	> 0.05
SHAPS	**5.67**	2.39	2.03	0.052	> 0.05
SIGH	**21.33**	10.78	10.39	0.047	> 0.05
STAIS	**51.0**	46.32	44.66	0.021	> 0.05
STAIT	**55.67**	48.91	45.34	0.056	> 0.05
YMRS	0.33	**3.83**	2.14	0.036	> 0.05

For the means (M) the mean of the group (LH: left-handers; MH: mixed-handers; RH: right-handers) with the highest or lowest value is marked in bold. For interpreting effect sizes, the upper and lower bounds given for Pearson’s r according to Cohen (1988) were squared. Accordingly, effect sizes were characterized as small ε^2^ = +/- 0.01 - +/- 0.09), medium (ε^2^ = +/−0.09 - +/- 0.25) and large (ε^2^ = +/- 0.25). * p < 0.05 (uncorrected), ** p < 0.01

## Discussion

The present study investigated associations between diagnosis, symptom severity, and hemispheric asymmetries (as indexed by handedness) in a large sample of patients suffering from affective disorders, schizoaffective disorders, or schizophrenia. Handedness scores were correlated with measures of symptom severity within and across different patient groups to test three possible types of associations between atypical hemispheric asymmetries and psychopathology: non-specific, diagnosis-specific, and symptom-specific associations. Across a series of complementary analyses, evidence in favor of non-specific and symptom-specific associations between non-right-handedness and symptoms emerged despite large-scale studies and meta-analyses on hemispheric asymmetries that report only minimal overlap between disorders in structural hemispheric asymmetries [16, 22, 65].

First, patients generally demonstrated a higher rate of mixed-handedness (15.7%) compared to healthy controls (12%), independent of diagnosis. In comparison, around 9.3% of the general population are mixed-handed [66]. This increased rate of mixed-handedness rates among patients, irrespective of diagnosis, is in line with previous findings of increased rates of mixed- or non-right-handedness in patients with posttraumatic stress disorder [10] and increased rates of non-right-handedness in patients with schizophrenia [11]. In contrast to a recent meta-analysis [39], increased rates of mixed-handedness in patients with MDD were also found.

Second, when analyzing the correlation coefficients for LQ and measures of symptom severity in patients independent of diagnosis, no association between handedness and symptom severity was found. The same was true in healthy individuals.

Third, patients with schizophrenia demonstrated the most differences in symptom severity between left-, mixed- and right-handers, with left-handed patients demonstrating overall higher symptom severity. Left-handers with schizophrenia showed the highest symptom scores on almost all measures. Moreover, in patients with schizophrenia and patients with schizoaffective disorders, symptom scores did not only differ between handedness groups for disorder-specific measures, such as positive and negative symptoms, paranoia, or mania, but also for less directly related symptoms, such as depression, anxiety, and somatization. These findings support both a symptom-specific and a diagnosis-specific association in patients with schizophrenia with more severe symptoms associated with increased non-right-handedness. Similar associations have already been reported by others in patients with schizophrenia [41–43], lending additional support to the assumption of a symptom-related atypical hand preference in schizophrenia. The relevance of symptom severity for the association between symptoms and atypical hemispheric asymmetries has also been shown in animal models of mental disorders. Here, specific symptoms of depression, such as anhedonia or behavioral despair, were significantly increased in left-lateralized animals, and left-lateralized behavior even had a predictive value of symptom severity [36–38].

The reported associations between handedness and symptom severity in schizophrenia and schizoaffective disorders suggest symptom-specific distinctions in how asymmetries may relate to symptomatology. One explanation Hugdahl and colleagues have proposed is a specific functional overlap between symptoms and asymmetry [67]. In brief, they suggest that auditory hallucinations may be thought of as internally generated speech perceptions lateralized to the left temporal lobe that result from left-lateralized alterations [67]. This is reflected in reduced left temporal lobe grey matter density in hallucinating patients, along with a failure to demonstrate an expected right ear advantage on the dichotic listening test in patients with schizophrenia [67]. Aligned with these results, meta-analyses have revealed that especially hallucinating patients with schizophrenia demonstrate a stronger reduction of language lateralization than non-hallucinating controls [68]. In our study, negative effect sizes were found for the scales “Hallucination” and for “Positive formal thought disorder”, indicating higher symptom severity in schizophrenia patients with an increased tendency towards left-handedness. Since left-handed individuals are more likely to show atypical right-sided language lateralization [69], this finding suggests that handedness may be a relevant factor for the relation of schizophrenia and language lateralization that should be considered in future studies.

The observed association between symptoms in schizophrenia and left-handedness could also partly be explained by genetics. A genome-wide association study found positive genetic correlations between left-handedness and traits of neuropsychiatric disorders such as schizophrenia and bipolar disorder [27]. As of yet, it seems most likely that several risk factors such as a genetic predisposition for non-right-handedness as well as for mental disorders, together with external influences and inherent atypical hemispheric asymmetries, may explain the association between handedness and psychopathology [70].

In addition, we tested whether familial predisposition (defined as one or more first-degree relatives having a psychiatric diagnosis) and early life events affect handedness. However, both do not seem to have a substantial influence independent of diagnosis, which aligns with results from studies in healthy participants [2, 3].

Generally, it is important to extend the assessment of behavioral asymmetries (i.e., handedness) to hand performance tests (e.g., Pegboard Test [71, 72]) as well as the observation of real-life side biases such as maternal cradling [73, 74] and embracing [75] in future cohort studies, enabling an even more detailed comprehension of the association between hemispheric asymmetries and clinical conditions.

However, some methodological limitations of the present study have to be considered before any final conclusion about the association between diagnosis, symptom severity, and handedness can be drawn. First, the analyses may have been affected by small sample sizes for each diagnosis, small effect sizes, and multiple comparisons, so results should be interpreted cautiously. Second, the analyses may also have been affected by unequal numbers of left-, right-, and mixed-handers in the respective samples. However, left- and mixed-handers are generally relatively rare in the population [66], rendering the present sample quite representative of the respective populations. Third, only some of the most common mental disorders were considered in the analyses, which limits the generalizability of the present findings. Fourth, we did not control for comorbid disorders but only considered the primary diagnosis for categorizing patients. Also, our analyses are cross-sectional and thus do not allow to draw causal conclusions. Finally, results rely on self-reporting hand preference and no objective measurements of left- and right-hand skills. Some symptoms might affect the ability to monitor one’s preferences, which could explain some results. Future studies investigating handedness in larger samples of patients showing a more comprehensive range of symptoms across more mental disorders are needed to elucidate associations between symptom severity and handedness further.

Moreover, future interventional studies could make major contributions to the understanding of hemispheric asymmetries in clinical groups. On the one hand, this could include the repeated assessment of handedness and other forms of hemispheric asymmetries in randomized clinical trial studies to assess whether a treatment that improves the clinical condition also affects hemispheric asymmetries. On the other hand, neuromodulatory techniques such as transcranial magnetic stimulation could be used to modulate functional hemispheric asymmetries in order to assess the impact of such interventions on symptom severity.

Overall, the present study showed that patients, irrespective of their specific disorders, showed a general increase in rates of mixed-handedness compared to healthy controls. Taking patients’ specific diagnoses into account, this increase in mixed-handedness was particularly pronounced among patients with schizophrenia, followed by patients with MDD. Regarding symptom-specific associations, left-handed patients with schizophrenia demonstrated the highest symptom severity. There was no pattern evident across disorders. The present results suggest that both a non-specific association between non-right-handedness (as an index of atypical hemispheric asymmetry) and psychopathology in mental disorders and a symptom-specific association within patients with schizophrenia are evident.

## Data Availability

Data sharing does not apply to this article as no new data were created or analyzed in this study. All data analyzed in the present study were taken from the FOR2107 study.

## Abbreviations

BDI: Beck Depression Inventory
BD: Bipolar disorder
CTQ: Childhood Trauma Questionnaire
EHI: Edinburgh Handedness Inventory
GAF: Global Assessment of Functioning scale
HAMA: Hamilton Anxiety Scale
HAMD: Hamilton Rating Scale for Depression
LH: Left-hander
LQ: Lateralization quotient
MH: Mixed-hander
MDD: Major Depressive Disorder
PSS: Perceived Stress Scale
RH: Right-hander
SANS: Scale for the Assessment of Negative Symptoms
SAPS: Scale for the Assessment of Positive Symptoms
SCID: Structured Clinical Interview for DSM
SCL-90-R: Symptom Checklist-90-Revised
SHAPS: Snaith-Hamilton-Pleasure Scale
SIGH: Structured Interview Guide for the Hamilton depression rating scale
STAI: State-trait Anxiety Inventory
YMRS: Young Mania Rating Scale
